# Characterization of the m^6^A regulator-mediated methylation modification patterns in oral squamous cell carcinoma

**DOI:** 10.1038/s41598-023-33891-9

**Published:** 2023-04-24

**Authors:** Lu Pan, He She, Keyi Wang, Wenhui Xia, Haonan Tang, Yuan Fan, Jinhai Ye

**Affiliations:** 1 Department of Oral Mucosal Diseases, The Affiliated Stomatological Hospital of Nanjing Medical University, 136# Hanzhong Road, Nanjing, 210000 Jiangsu China; 2 Department of Oral and Maxillofacial Surgery, The Affiliated Stomatological Hospital of Nanjing Medical University, 136# Hanzhong Road, Nanjing, 210000 Jiangsu China; 3 Jiangsu Province Key Laboratory of Oral Diseases, Nanjing Medical University, Jiangsu, China; 4 Jiangsu Province Engineering Research Center of Stomatological Translational Medicine, Jiangsu, China

**Keywords:** Cancer therapy, Oral cancer

## Abstract

*N*^6^-methyladenosine (m^6^A) is a form of posttranscriptional modification that plays important roles in cancer including oral squamous cell carcinoma (OSCC). Most studies to date have focused on a limited number of regulators and oncogenic pathways, thus failing to provide comprehensive insight into the dynamic effects of m^6^A modification. In addition, the role of m^6^A modification in shaping immune cell infiltration in OSCC has yet to be clarified. This study was designed to assess m^6^A modification dynamics in OSCC and to understand how such modifications influence clinical immunotherapeutic treatment outcomes. m^6^A modification patterns linked with 23 m^6^A regulators were analyzed in 437 OSCC patients from TCGA and GEO cohorts. These patterns were then quantified through m^6^A score based on algorithms derived from a principal component analysis (PCA) approach. The m^6^A modification patterns of OSCC samples were grouped into two clusters based on the m^6^A regulators expression, and immune cell infiltration was linked with the 5-year survival outcomes of patients in these clusters. 1575 genes associated with OSCC patient prognosis were identified and used to re-cluster these samples into two groups. Patients in clusters exhibiting higher levels of m^6^A regulator expression exhibited poorer overall survival (OS), whereas patients with high m^6^A scores survived for longer (p < 0.001). The overall mortality rates in the groups of patients with low and high m^6^A scores were 55% and 40%, respectively, and the m^6^A score distributions in clusters of patients grouped by m^6^A modification patterns and gene expression further supported the link between a high m^6^A score and better prognostic outcomes. Immunophenoscore (IPS) values for patients in different m^6^A score groups suggested that the use of PD-1-specific antibodies or CTLA-4 inhibitors alone or in combination would yield superior treatment outcomes in patients in the high-m^6^A score group relative to the low-m^6^A score group. m^6^A modification patterns are relevant to heterogeneity in OSCC. Detailed analyses of m^6^A modification patterns may thus offer novel insight regarding immune cell infiltration within the OSCC tumor microenvironment, guiding novel efforts to provide patients with more effective immunotherapeutic interventions.

## Introduction

Oral squamous cell carcinoma (OSCC) accounts for over 90% of all oral malignancies, and the 5-year overall survival (OS) rate for affected patients is just 60%^1^. While there have been advances in the diagnosis and treatment of OSCC patients in recent years, this has failed to translate to a pronounced increase in OS, with tumor immune evasion playing an important role in poor patient outcomes^2^. The advent of immune checkpoint inhibitors (ICIs) has offered a new opportunity to treat OSCC, with anti-PD-1/PD-L1 immunotherapeutic regimens having demonstrated efficacy in advanced head and neck squamous cell carcinoma cases^3^. However, only an estimated 20–40% of patients ultimately benefit from ICI administration^4^. Treating OSCC thus remains a difficult clinical challenge, and the identification of reliable biomarkers associated with patient responses to immunotherapeutic treatment and prognostic outcomes has the opportunity to reduce the morbidity of OSCC by providing patients with more personalized and effective pharmacological tools.

The *N*^6^-methyladenosine (m^6^A) modification of mRNA is the most common posttranscriptional modification in eukaryotic cells, shaping a range of physiological and pathogenic processes^5^. A diverse array of methyltransferases, demethylases, and m^6^A binding proteins (respectively known as “writers”, “erasers”, and “readers) shape the m^6^A methylation landscape in a dynamic manner. When these m^6^A regulatory proteins are dysregulated, this can alter the expression of oncogenes in a manner that may ultimately be conducive to oncogenic transformation^6^. For example, in tumors from acute myeloid leukemia (AML) patients, significant increases in the expression of METTL3, METTL14, and YTHDF2 have been reported^7^. In lung adenocarcinoma, METTL3 can promote the enhanced translation of oncogenic factors including BRD4, EGFR, and TAZ in cooperation with EIF3^8,9^. Other m^6^A regulators that have been studied to date include IGF2BP1, YTHDF1, YTHDF2, and FTO^6^. High-level alterations in global m^6^A abundance have been linked to tumor progression, metastasis, chemoresistance, and recurrence^10^. However, a majority of studies conducted to date have only focused on a limited number of m^6^A regulators and oncogenic pathways without fully exploring the complex and dynamic m^6^A modification landscape, thus failing to provide a comprehensive overview of how this form of posttranscriptional modification shapes pro-tumorigenic processes. There have also been several reports that m^6^A modification plays a role in shaping the composition of the tumor-associated immune microenvironment and related immune response induction^11,12^, although the precise mechanisms underlying such activity are poorly understood, particularly in OSCC. As such, further detailed studies of a variety of m^6^A regulatory proteins may offer new insight regarding the role that m^6^A modification plays in the pathogenesis of OSCC. These analyses may further enable the identification of distinct subgroups of OSCC patients with specific tumor characteristics and immunophenotypes, thereby supporting efforts to define novel biomarkers that can predict patient responses to immunotherapeutic treatment.

The present study was developed to conduct a comprehensive analysis of OSCC-related patterns of m^6^A modification by integrating data from the Cancer Genome Atlas (TCGA) and Gene-Expression Omnibus (GEO) databases. Together, these analyses offered new insight into the dynamic landscape of m^6^A modification patterns and their relationship with intratumoral immune cell infiltration. In addition, these results were used to establish a scoring system to enable the quantification of m^6^A methylation levels in individual patient samples based on patterns of differentially expressed genes (DEGs) under different patterns. Immunophenoscore (IPS) values were further leveraged to guide the selection of appropriate immunotherapeutic interventions.

## Results

### Patterns of m^6^A regulator gene expression in OSCC

**Figure 1:**
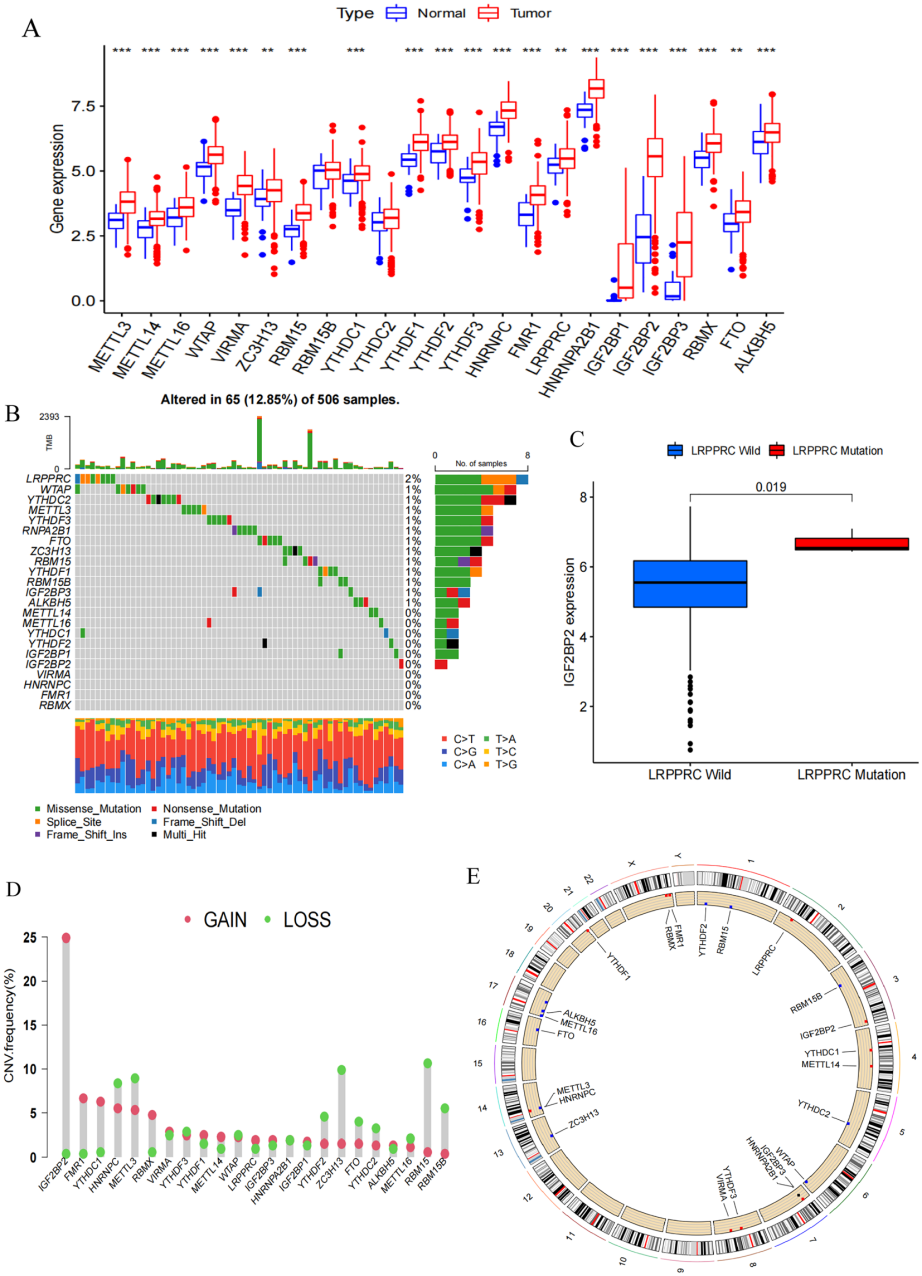
Landscape of genetic and expression variation of m^6^A regulators in OSCC. (**A**) Expression of 23 m^6^A regulator genes between normal tissues and OSCC tissues. Tumor, red; Normal, blue. Top edge of box: upper quartile; bottom edge of box: lower quartile; Internal horizontal line: median. Dots above and below: outliers. *p < 0.05; **p < 0.01; ***p < 0.001. (**B**) Mutation frequency of 23 m^6^A regulator genes in 506 OSCC samples from TCGA database. Each column represented a sample. The upper barplot showed TMB; the lower barplot showed conversion fraction in each sample; the right barplot showed the proportion of each variant type. Number on the right represented the mutation frequency of each regulator gene. (**C**) Differential expression of IGF2BP2 in LRPPRC wild and LRPPRC mutant samples. LRPPRC mutant, red; LRPPRC wild, blue. Top edge of box: upper quartile; bottom edge of box: lower quartile; Internal horizontal line: median. Dots above and below: outliers. The upper number represented p value. (**D**) The CNV frequency of 23 m^6^A regulator genes in TCGA-HNSC cohort. The height of the column represented the CNV frequency. The deletion frequency, blue dot; the amplification frequency, red dot. (**E**) The CNV location of 23 m^6^A regulator genes on chromosomes.

An initial analysis was used to explore patterns of m^6^A regulator gene expression in OSCC patients and normal tissue samples (Fig. 1A), revealing that all of these genes were expressed at higher levels in OSCC tumor tissue samples consistent with the enhancement of m^6^A modification activity in OSCC. These differences were significant for all m^6^A regulator genes other than RBM15B and YTHDC2. The m^6^A regulator that exhibited the lowest levels of expression was IGF2BP1, whereas HNRNPA2B1 expression levels were highest. Next, the mutational status of these m^6^A regulators was assessed in OSCC samples, revealing that 65/506 samples (12.85%) harbored mutations in m^6^A regulator genes (Fig. 1B). These mutations were spread across 13 m^6^A regulator genes, of which LRPPRC exhibited the highest mutational frequency. Patients harboring LRPPRC mutations also expressed higher levels of IGF2BP2 (Fig. 1C). These mutation and expression data are consistent with a model wherein interactions among m^6^A regulators, rather than individual regulatory proteins, ultimately shape the development and pathogenesis of OSCC such that combination treatment strategies will be critical to effectively remediate dysregulated m^6^A modification patterns in this oncogenic context. Copy number variations (CNVs) were also observed for all 23 m^6^A regulator genes in this patient cohort (Fig. 1D). Copy number amplifications were the primary CNVs observed for 11 of these genes (IGF2BP2, FMR1, YTHDC1, RBMX, VIRMA, YTHDF1, METTL14, LRPPRC, IGF2BP3, IGF2BP1, and ALKBH5), whereas the remainder primarily presented with copy number losses. The locations of these m^6^A CNVs were also noted on specific chromosomes (Fig. 1E). Together, these results suggest that OSCC tumors exhibit increased m^6^A regulator gene expression relative to normal tissues, and that mutations in these m^6^A regulator genes are a common finding in OSCC consistent with the important role that m^6^A methylation modification plays as a driver of tumor malignancy and progression.

### Analyses of the prognostic relevance of individual m^6^A regulators

**Figure 2:**
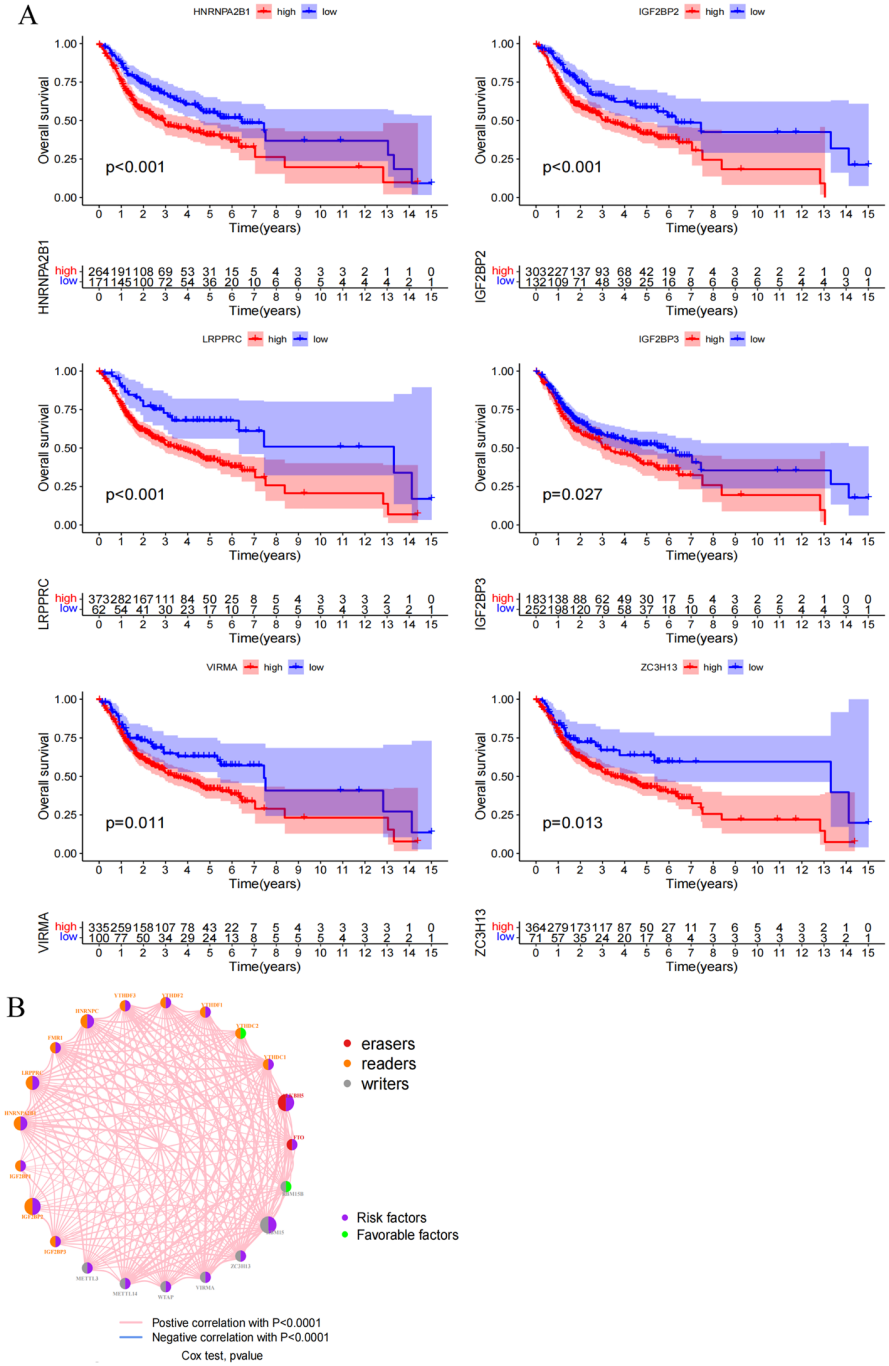
Correlation of m^6^A regulators with prognosis. (**A**) Kaplan–Meier survival analysis of m^6^A regulators between the high-expression group and the low-expression group using clinical information of OSCC patients in TCGA and GSE41613 cohort. High-expression, red curve; Low-expression, blue curve. P value less than 0.05 was statistically significant. (**B**) The interaction between m^6^A regulators in OSCC. Writers, readers and erasers were marked with red, orange and gray, respectively. The circle size represented the effect of each regulator on prognosis, and the range of values calculated by Cox test was p < 1e−04, p < 0.001, p < 0.01, p < 0.05 and p < 1, respectively. Green in the circle, favorable factors of prognosis; Purple in the circle, risk factors of prognosis. Curves linking regulators showed their interactions, with thickness showing the correlation strength. Positive correlation with p < 0.0001, pink curve; Negative correlation with p < 0.0001, blue curve. The figure was generated using R software (V 4.1.2, https://www.r-project.org/).

To gain further insight into the association between m^6^A regulator genes and patient outcomes, survival outcome data were extracted after pooling the TCGA and GSE41613 datasets^13^. As the METTL16 and RBMX genes were not included in the GEO dataset, only the remaining 21 m^6^A genes were subject to subsequent analyses. Univariate Cox regression models (Table 1) indicated that RBM15, HNRNPC, LRPPRC, HNRNPA2B1, IGF2BP2, and ALKBH5 were associated with a high risk of worse patient survival (p < 0.05), while only two genes (RBM15B, YTHDC2) were associated with lower risk. Patients were then stratified into two groups based on whether they expressed high or low levels of each of these genes, after which Kaplan–Meier OS curves were generated. Of the 15 m^6^A regulator genes associated with patient OS in these analyses, HNRNPA2B1, IGF2BP2, IGF2BP3, LPPPRC, VIRMA, and ZC3H13 were found to be associated with poor prognostic outcomes (Fig. 2A, Fig. S1). To more fully explore the interconnected relationships among these genes and the prognostic relevance of these m^6^A regulators, a network diagram was constructed based on correlative relationships (p < 0.0001) (Fig. 2B). The expression of m^6^A regulators included in the same functional category was found to be positively correlated, and positive correlations were also observed among these m^6^A writers, readers, and erasers. These data thus indicate that m^6^A regulators can be used to predict prognostic outcomes in individuals with OSCC, with interactions among these three classes of regulators potentially playing a critical role in key oncogenic processes.

**Table 1:** Univariate Cox regression analyses of 21 m^6^A regulator genes.

Id	HR	HR.95L	HR.95H	p value
METTL3	1.116450541	0.887880525	1.403862091	0.345941334
METTL14	1.294120728	0.953839554	1.755796824	0.097649322
WTAP	1.275904913	0.961740163	1.692695605	0.091129712
VIRMA	1.256920165	0.980807765	1.610762432	0.070787468
ZC3H13	1.151299409	0.930786384	1.424054275	0.194020397
RBM15	1.538514029	1.12859059	2.097328685	0.006426907
RBM15B	0.800297158	0.60596148	1.056957516	0.116495641
YTHDC1	1.11875648	0.838479327	1.492721431	0.445656819
YTHDC2	0.926771542	0.740830915	1.159381275	0.505664518
YTHDF1	1.216982269	0.889451958	1.665121797	0.219591951
YTHDF2	1.151330029	0.831208707	1.594738871	0.396571181
YTHDF3	1.124648663	0.908172936	1.39272441	0.281511088
HNRNPC	1.471383559	1.085258088	1.994889143	0.012889922
FMR1	1.015795476	0.802626295	1.285580171	0.896241771
LRPPRC	1.321550101	1.038467418	1.681800161	0.023398623
HNRNPA2B1	1.345859165	1.02644532	1.764669638	0.031651178
IGF2BP1	1.002697307	0.89487228	1.123514396	0.962986711
IGF2BP2	1.206651307	1.058843373	1.375092307	0.004838834
IGF2BP3	1.056608342	0.95945766	1.163596096	0.263163273
FTO	1.113614571	0.905564814	1.369462895	0.307796808
ALKBH5	1.528065039	1.158427783	2.015648105	0.002692969

HR value > 1: high-risk gene; HR value < 1: low-risk gene. p < 0.05 was statistically significant.

### m^6^A methylation modification pattern characteristics

**Figure 3:**
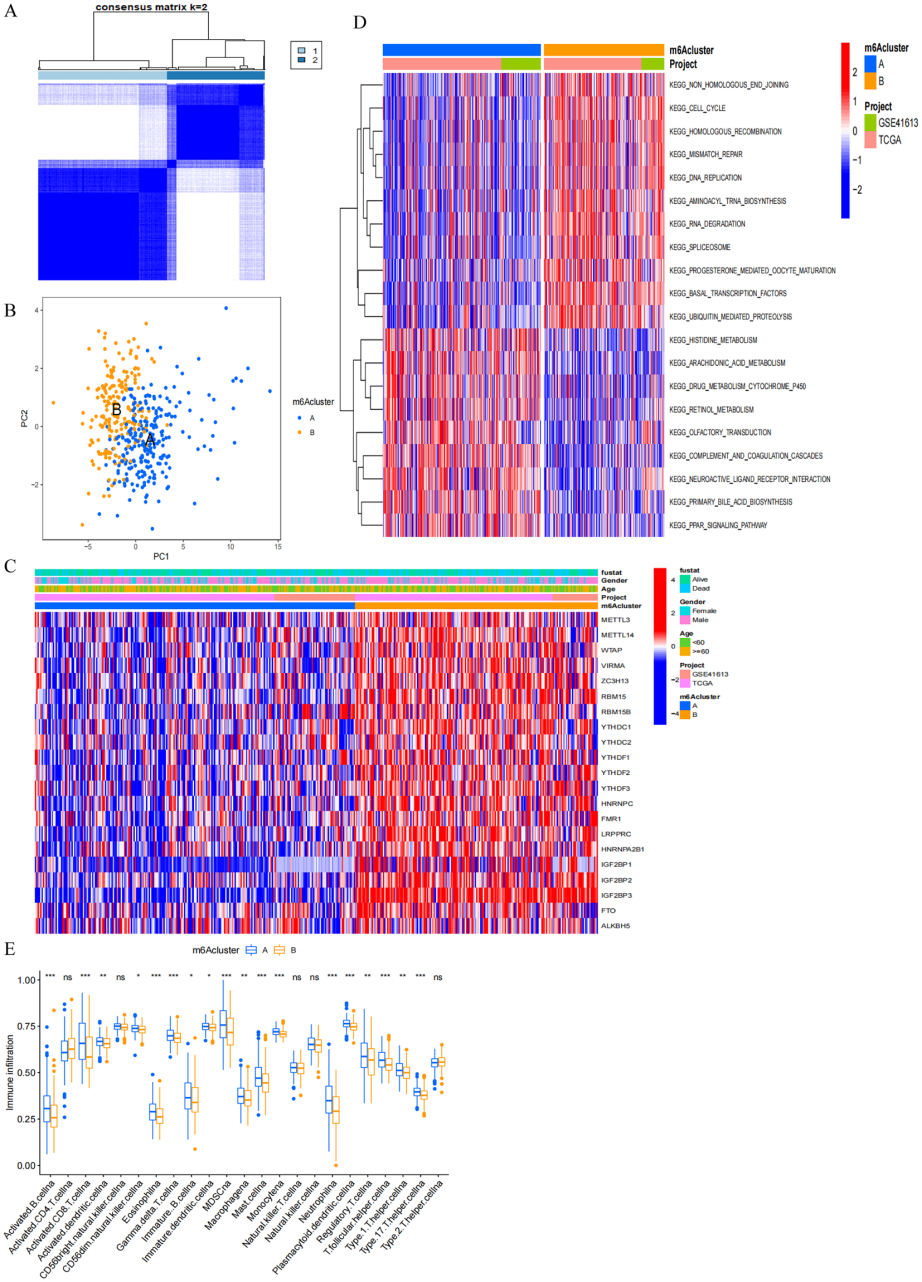
Characteristics of m^6^A methylation modification patterns. (**A**) Consensus clustering analysis of m^6^A regulators. Consensus clustering matrix for k = 2. Cluster A, 1; Cluster B, 2. (**B**) PCA analysis of m^6^A regulators. Samples in cluster A, blue dots; Samples in cluster B, yellow dots. (**C**) Unsupervised clustering of m^6^A regulators in OSCC patients from TCGA and GSE41613 cohort. Fustat, gender, age, project and m^6^Acluster were used as patient annotations. High expression, red; low expression, blue. (**D**) The heatmap of GSVA showing KEGG pathways in each m^6^A modification patterns. Red represented activated pathways and blue represented inhibited pathways. KEGG pathways^14–16^ were downloaded from the website (http://www.gsea-msigdb.org/). (**E**) ssGSEA of immune cells infiltration in individual m^6^A modification patterns. Cluster A, bule; Cluster B, yellow. Top edge of box: upper quartile; bottom edge of box: lower quartile; Internal horizontal line: median. Dots above and below: outliers. *p < 0.05; **p < 0.01; ***p < 0.001; *ns* no statistical significance.

Next, the R “ConsensusCluster” package was used to classify patient tumor samples into two clusters based on the patterns of m^6^A regulator gene expression in these samples (Fig. 3A). PCA scatter plots revealed that samples within each of these clusters, designated cluster A and cluster B, were effectively grouped together (Fig. 3B). A heatmap analysis revealed that patterns of m^6^A regulator gene expression were markedly increased in cluster B (Fig. 3C). A non-significant trend towards better 5-year OS was observed in cluster A relative to cluster B (Fig. S2). These results were consistent with the ability of m^6^A regulators including VIRMA, ZC3H13, HNRNPA2B1, IGF2BP2, IGF2BP3, and LPPPRC to regulate mRNA m^6^A methylation patterns in OSCC in a manner that favors cancer progression. A GSVA approach was further used to explore differences in biological activity in these two sample clusters (Fig. 3D). Samples in cluster A exhibited pronounced metabolic pathway enrichment, whereas samples in cluster B were enriched for pathways associated with proliferation and DNA repair including the nonhomologous end joining, homologous recombination and mismatch repair, cell cycle, DNA replication, and aminoacyl tRNA biosynthesis pathways. These results further emphasize the differences in the biological characteristics of tumor samples from patients in these two m^6^A regulator-based subgroups. Intratumoral immune cell infiltration was next examined in these two clusters (Fig. 3E), revealing significant enrichment for various immune cell types in samples from cluster A, consistent with the better 5-year survival outcomes in this group that may be indicative of more robust antitumor immunity. Based on this combination of immune cell infiltration and prognostic results, cluster A was designated an immune-inflammatory phenotype, while cluster B was designated an immune-desert phenotype. These results further support the ability of m^6^A methylation modification patterns to impact antitumor immune responses.

### Identification of prognosis-related DEGs

**Figure 4:**
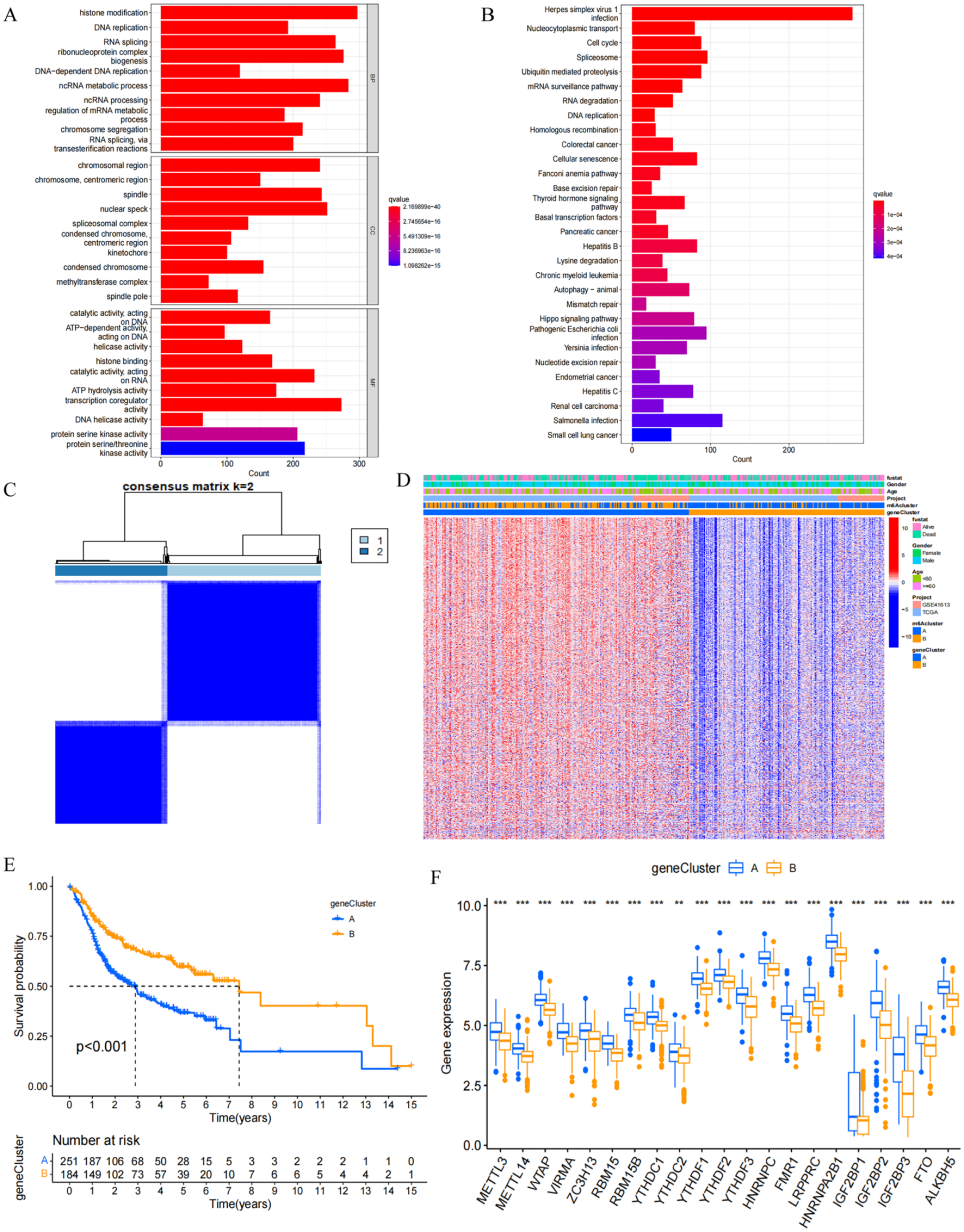
DEGs associated with prognosis. (**A**) Functional annotation of DEGs by GO enrichment analysis. The color depth of the barplot represented q value and the length of the barplot represented number of enriched genes. (**B**) Functional annotation of DEGs by KEGG enrichment analysis. The color depth of the barplot represented q value and the length of the barplot represented number of enriched genes. (**C**) Consensus clustering analysis of prognosis related genes. Consensus clustering matrix for k = 2. Gene cluster A, 1; Gene cluster B, 2. (**D**) Unsupervised clustering of prognosis related genes in OSCC patients from TCGA and GSE41613 cohort. Fustat, gender, age, project, m^6^Acluster and gene cluster were used as patient annotations. High expression, red; low expression, blue. (**E**) Kaplan–Meier survival analysis of OSCC patients in different gene clusters. Gene cluster A, blue curve; Gene cluster B, yellow curve. Log-rank p < 0.001 showed a significant survival difference between two gene clusters. (**F**) Differential expression of m^6^A regulators between gene cluster A and gene cluster B. Gene cluster A, blue; Gene cluster B, yellow. Top edge of box: upper quartile; bottom edge of box: lower quartile; internal horizontal line: median. Dots above and below: outliers. *p < 0.05; **p < 0.01; ***p < 0.001.

Next, DEGs associated with m^6^A modification patterns were identified, yielding 7075 total genes. GO enrichment analyses of these DEGs were then performed for three major categories of GO terms (Fig. 4A), and KEGG enrichment analyses revealed these genes to be associated with oncogenesis and infection-related pathways (Fig. 4B). Univariate Cox regression analysis screening then identified 1575 prognosis-related genes, of which 4% (64/1575) were associated with lower risk, while the remainder were associated with increased risk (p < 0.05). The top 10 low- and high-risk genes are presented in Table S1. These 1575 genes were then used to repeat the clustering of OSCC patient samples into two clusters (gene cluster A and gene cluster B) (Fig. 4C). Heatmap analyses indicated that these two gene clusters exhibited distinct gene expression signatures (Fig. 4D). Kaplan–Meier analyses revealed that patients in gene cluster B exhibited increased OS as compared to patients in gene cluster A (p < 0.001) (Fig. 4E). Patients in gene cluster A also exhibited significant increases in the expression of all m^6^A regulator genes (Fig. 4F). These data confirmed that m^6^A methylation modifications are associated with antitumor immunity.

### Higher m^6^A score values are linked with better prognostic outcomes

**Figure 5:**
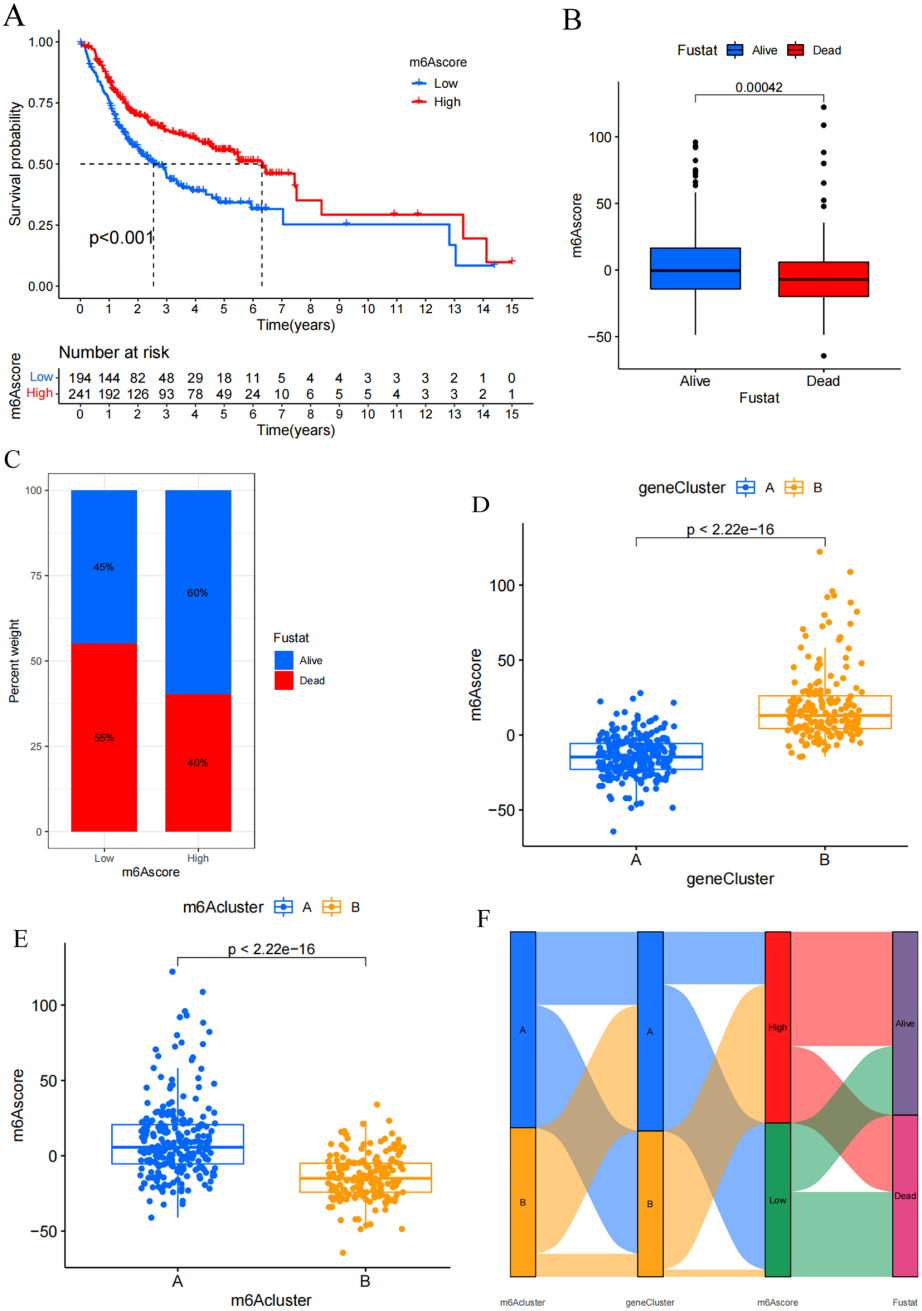
High m^6^A score associated with better prognosis. (**A**) Kaplan–Meier survival analysis of OSCC patients in different m^6^A score groups. Low-m^6^A score group, blue curve; High-m^6^A score group, red curve. Log-rank p < 0.001 showed a significant survival difference between two groups. (**B**) Differences in m^6^A score between alive and dead patients in TCGA and GSE41613 cohort. Alive, blue; Dead, red. Top edge of box: upper quartile; bottom edge of box: lower quartile; internal horizontal line: median. Dots above and below: outliers. The upper number represented p value. (**C**) The proportion of alive and dead patients in the low-m^6^A score group and the high-m^6^A score group. Alive, blue; Dead, red. Numbers in the barplots represented percentages. (**D**) Differences in m^6^A score between m^6^A clusters. Cluster A, blue; Cluster B, yellow. Top edge of box: upper quartile; bottom edge of box: lower quartile; internal horizontal line: median. Dots represented samples and the upper number represented p value. (**E**) Differences in m^6^A score between gene clusters. Gene cluster A, blue; Gene cluster B, yellow. Top edge of box: upper quartile; bottom edge of box: lower quartile; internal horizontal line: median. Dots represented samples and the upper number represented p value. (**F**) Sankey diagram showing the correlations among m^6^A clusters, gene clusters, m^6^A score and fustat.

To better classify OSCC patients and to mitigate heterogeneity among these patients, a scoring system was next established to quantify patterns of m^6^A methylation termed the m^6^A score, which was based on these 1575 prognosis-associated genes. An optimal m^6^A score cutoff was established with the “survminer” package, with patients then being stratified into high- and low-m^6^A score groups. Individuals in the high-m^6^A score group exhibited significantly better survival than patients in the low-m^6^A score group (p < 0.001) (Fig. 5A). Box-plot analyses revealed that the median m^6^A score values were higher in living patients than in deceased patients (Fig. 5B). The overall mortality rates in the low- and high-m^6^A score groups were 55% and 40%, respectively (Fig. 5C). These data indicate that higher m^6^A scores are related to better prognostic outcomes. Further analyses were conducted comparing correlations between OS, m^6^A score values, and clinical T stage (Fig. S3). No significant differences were observed for stages T1–2 or stages T3–4. Moreover, score distributions were calculated for patients in different clusters generated based on m^6^A methylation modification patterns and gene clusters, revealing that in both settings the patient clusters that exhibited higher survival rates also exhibited higher m^6^A scores (Fig. 5D,E). Sankey diagrams were further used to visualize these results (Fig. 5F). In general, patients in the high-m^6^A score group were primarily individuals from gene cluster B with better prognostic outcomes, as well as some patients in m^6^A cluster A with longer OS. As such, these findings indicate that m^6^A score can be used to predict OSCC patient clinical outcomes.

### The relationship between mutational burden, immunity, and tumor m^6^A modification

**Figure 6:**
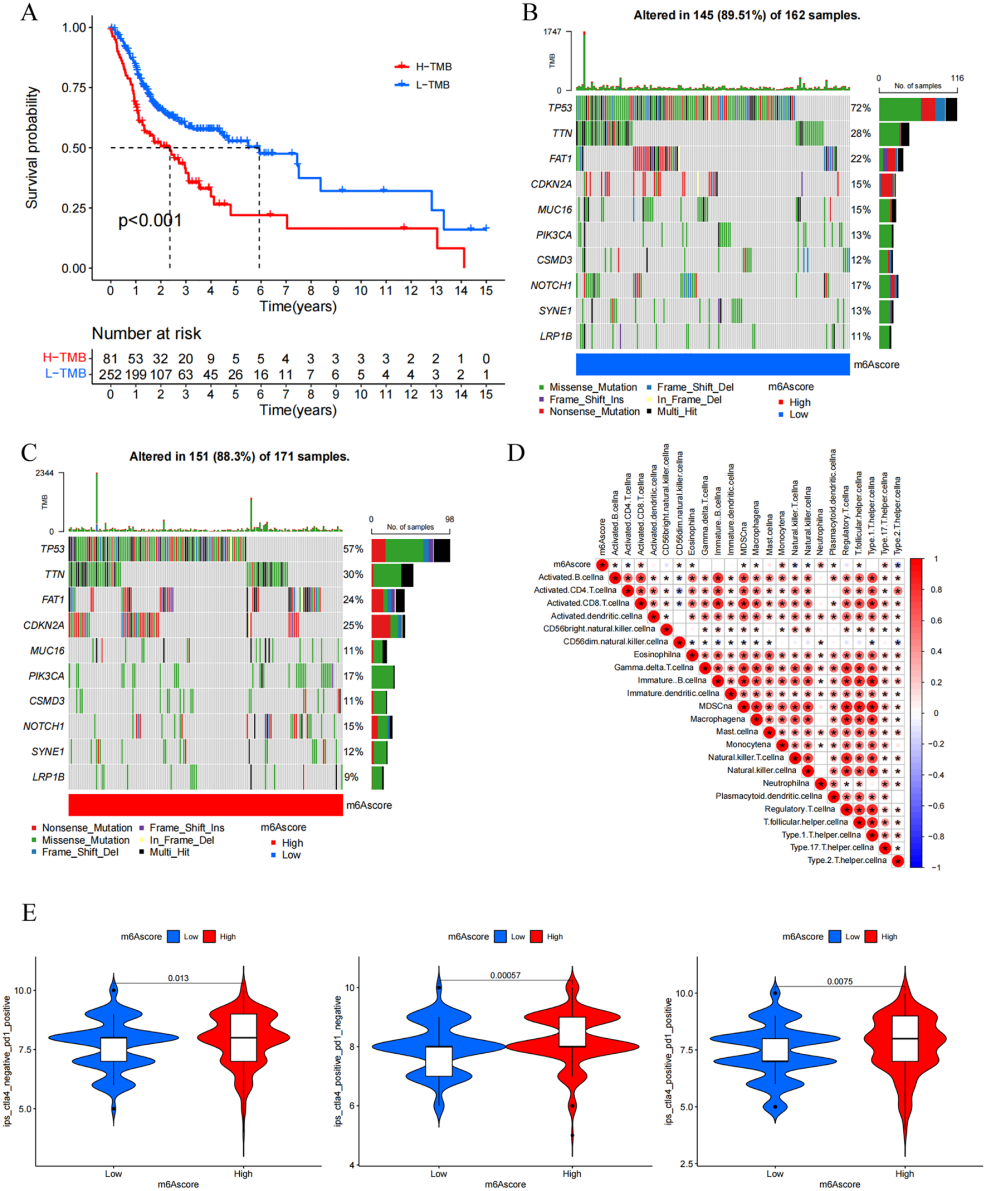
Mutation and immunity in tumor m^6^A modification patterns. (**A**) Kaplan–Meier survival analysis of OSCC patients in low TMB group and high TMB group. Low TMB group, blue curve; High TMB group, red curve. Log-rank p < 0.001 showed a significant survival difference between two groups. (**B,C**) TMB of top ten high-frequency mutated genes in 162 samples from low-m6Ascore group (**B**) and 171 samples from high-m6Ascore group (**C**). Each column represented a sample. The upper barplot showed TMB; the right barplot showed the proportion of each variant type. Numbers on the right represented the mutation frequency of genes. (**D**) Correlations between m^6^A score and immune cells infiltration using Spearman analysis. Negative correlation was marked with blue and positive correlation with red. *p < 0.05. (**E**) Correlations between m^6^A score and IPS. Low-m^6^A score group, blue; High-m^6^A score group, red. The upper number represented p value. p value less than 0.05 was statistically significant.

An increased tumor mutational burden (TMB) entails neoantigen production such that these antigens can be recognized by T cells, thereby promoting immune response activation^17^. Next, the OS of patients with differing TMB frequencies was compared (Fig. 6A). In contrast to literature-based expectations, the survival of patients in the low TMB group was actually higher than that of patients in the high TMB group. No significant differences in TMB were observed when comparing the low- and high-m^6^A score groups (Fig. S4). This suggests that there may not be any significant relationship between TMB and m^6^A modification with respect to OSCC patient outcomes. The R “maftools” package was then used to explore differences in somatic mutations between individuals in the low- and high-m^6^A score groups based on the ten most frequently mutated genes (Table S2). Similar mutational frequencies for these ten genes were observed in both groups (89.51% vs. 88.30%) (Fig. 6B,C), although a 1.3-fold higher TP53 mutation rate was observed in the low-m^6^A score group relative to the high-m^6^A score group (72% vs. 57%), and the CDKN2A mutation rate in the high-m^6^A score group was 1.7-fold higher than in the low m^6^A score group (25% vs. 15%). As such, while overall TMB was not related to patient prognosis, a relationship was observed between TP53 and CDKN2A mutation status and m^6^A modification-related differences in patient prognosis.

Correlations between m^6^A score and immune cell infiltration were next examined (Fig. 6D), revealing that 11 immune cell types were positively correlated with m^6^A score, whereas activated CD4^+^ T cells, NK T cells, NK cells, and Th2 cells were negatively correlated with m^6^A score. The expression of immune checkpoint genes was also examined (Fig. S5), but no significant differences in PD-L1 or CTLA-4 expression were observed. To explore the efficacy of immunotherapeutic treatment, patient IPS scores were examined (Fig. 6E). These scores suggested that individuals in the high-m^6^A score group would experience superior outcomes relative to patients in the low-m^6^A score group irrespective of whether they were treated with PD-1-specific antibodies, CTLA-4 inhibitors, or a combination of both of these ICIs. In addition, the IPS distributions in the high-m^6^A score group were more concentrated when patients were treated with PD-1-specific antibodies alone or in combination with CTLA-4 inhibitors as compared to CTLA-4 inhibitors alone, while the combination of PD-1-specific antibodies and CTLA-4 inhibitors was not more effective. These findings suggest that m^6^A score can predict OSCC patient immunotherapy responses as compared to traditional analyses of TMB, with patients with higher m^6^A scores being more sensitive to ICIs targeting PD-1 or CTLA-4 as compared to individuals with low m^6^A scores. However, individual heterogeneity was found to significantly impact the predicted efficacy of CTLA-4 inhibitor treatment.

## Discussion

Many recent studies have examined the relationship between m^6^A modifications and cancer, underscoring the importance of such posttranscriptional modifications in OSCC. However, these prior studies have focused only on specific individual regulators such as METTL3^18–20^, METTL14^21^, ALKBH5^22^, FTO^23,24^, and HNRNPA2B1^25^. How other m^6^A-related regulatory proteins shape the pathogenesis of OSCC remains to be established. In addition, the different classes of m^6^A readers, writers, and erasers do not function independently of one another, instead interacting in a dynamic manner to govern patterns of m^6^A methylation. To date, technological limitations have hampered efforts to comprehensively evaluate these different factors. As such, a bioinformatics approach was herein used to examine as many regulatory factors as possible with the goal of better understanding the importance of different m^6^A regulatory mechanisms in OSCC, thereby providing a foundation for future work.

In these analyses, RBM15, HNRNPC, LRPPRC, HNRNPA2B1, IGF2BP2, and ALKBH5 were identified as high-risk genes, whereas HNRNPA2B1, IGF2BP2, IGF2BP3, LPPPRC, VIRMA, and ZC3H13 were related to poorer prognostic outcomes, suggesting that HNRNPA2B1, IGF2BP2, and LPPPRC are particularly reliable predictors of negative patient outcomes. Higher HNRNPA2B1 levels in individuals with OSCC have previously been reported to be associated with poorer OS^25^. HNRNPA2B1 can also contribute to the progression of a range of solid and hematological tumor types. Jiang et al.^26^ further determined that HNRNPA2B1 was upregulated in myeloma and linked to poorer outcomes through its ability to stabilize the ILF3 mRNA and promote AKT3 upregulation. IGF2BP2 has also been reported to be related to patient outcomes in individuals with colorectal cancer^27^, hepatocellular carcinoma^28^, pancreatic cancer^29^, gastric cancer^30^, and OSCC^31^, while LPPPRC is an independent predictor of prognostic outcomes in many cancers. While these results support the present findings, research focused on HNRNPA2B1 and IGF2BP2 in OSCC has been limited, and no studies have assessed LPPPRC in this oncogenic context. As such, more work is needed to fully explore the prognostic relevance of these genes in OSCC.

Substantial heterogeneity is evident when comparing tumor cells among individuals with the same form of cancer, and this heterogeneity is also evident within tumors in a given patient^32^. Tumor complexity is often explained using a model outlining eight major hallmarks of cancer^33^, including the acquired capabilities for sustaining proliferative signaling, evading growth suppressors, resisting cell death, enabling replicative immortality, inducing/accessing vasculature, activating invasion and metastasis, reprogramming cellular metabolism, and avoiding immune destruction. In this study, OSCC patients were stratified into two groups based on patterns of m^6^A regulator expression, and the characteristics of patients in these clusters were assessed. While abundant immune cell infiltration was evident in cluster A together with the significant enrichment of metabolic pathways, cluster B exhibited a lack of immune cell infiltration together with enrichment for pathways associated with DNA repair and cellular proliferation. This suggests that high levels of m^6^A modification are related to certain hallmarks of cancer including resistance to cell death, supporting replicative immortality and the ability to evade immune-mediated destruction as observed for samples in m^6^A cluster B. While all OSCC tumors in this study are likely to exhibit these eight hallmarks of cancer to varying degrees, the actual associated phenotypes may vary among individuals, and m^6^A may shape these processes throughout the development and progression of OSCC given that the abilities to avoid apoptotic death and proliferate indefinitely may arise during different stages of oncogenesis. Different modification patterns may also impact prognostic outcomes given that individuals in gene cluster A exhibited the upregulation of all m^6^A regulator genes and such upregulation was related to poorer patient OS.

Univariate Cox regression analyses were further used to analyze DEGs identified by comparing these two patient clusters, focusing specifically on the ten highest and lowest risk genes. While their roles in OSCC remain to be studied in detail, some prior work does highlight potential mechanisms through which these genes may shape cancer patient outcomes. For example, in Ewing sarcoma, RING1B can repress SCN8A, thereby contributing to oncogenic progression in a manner independent of fusion oncoproteins^34^. Overexpression of CEACAM21, which is a member of the carcinoembryonic antigen family, has been detected in high-grade serous ovarian cancer in immune-activated tissues relative to immune-silent tissues^35^. SZT2 can suppress mTORC1 activity, thereby limiting the inhibitor effects of PI3Kα inhibitor and improving breast cancer-associated therapeutic efficacy^36^. In OSCC prognostic predictive models, NAGK has been identified as a low-risk gene^37^, while many high-risk genes have been studied in detail as oncogenes in other cancers^38–45^. Many of the top 10 low-risk genes identified herein have not been described previously in OSCC, and may thus offer novel predictive utility. In light of individual heterogeneity, m^6^A scores were utilized as a quantitative tool to compare patterns of m^6^A methylation among samples based on these prognostic genes. The resultant m^6^A scoring model has not previously been reported in OSCC, and was found to predict OSCC patient clinical outcomes such that higher m^6^A scores were linked to a better prognosis.

The majority of studies exploring m^6^A modification to date have analyzed oncogenic pathways without also examining the effects of such modifications on host antitumor immunity. However, a limited number of studies have examined the mechanisms through which specific m^6^A regulators can impact immune cell populations. For example, depleting METTL3 can enhance RIG-I-mediated innate immune responses to certain viruses^46^, while YTHDF2 is an m^6^A reader protein that regulates the maturation and homeostasis of NK cells, functioning as a positive regulator of antitumor immune responses^47^. YTHDF1 is capable of recognizing and enhancing the translation of m^6^A-modified lysosomal cathepsins in dendritic cells, thereby suppressing antigen cross-presentation and CD8^+^ T cell activation, ultimately inhibiting antitumor immunity^48^. In this study, significant differences in immune cell infiltration were evident when comparing patients in clusters A and B, and in KEGG analyses over 200 DEGs were enriched in the herpes simplex virus 1 infection pathway, supporting a link between differences in m^6^A modification and immune cell infiltration. High and low m^6^A score values were also related to different immune cell populations, emphasizing the important relationship between m^6^A modification and the composition of immune cells within the local microenvironmental landscape.

Important predictors of ICI responses identified to date include high levels of microsatellite instability, TMB, and the expression of CTLA-4 and PD-L1 by tumor cells^49^. The m^6^A scores generated herein were also identified as predictors of immunotherapeutic efficacy, with individuals in the high-m^6^A score group exhibiting greater predicted sensitivity to treatment with CTLA-4 inhibitors and PD-1-specific antibodies as compared to individuals in the low m^6^A score group. ICI treatment can facilitate tumor clearance by activating T cells and enabling them to more effectively recognize and respond to tumor-associated antigens. Higher TMB levels are associated with the generation of more neoantigens, thus providing these T cells with more opportunities to detect tumor cells, thus contributing to enhanced ICI efficacy^17^. In this study, however, patients with low TMB exhibited better OS outcomes, potentially because they did not undergo immunotherapeutic treatment. In patients not administered ICIs, higher TMB is associated with a poorer prognosis in many cancers^50^. While these neoantigens can aid in the establishment of spontaneous antitumor immunity, these immune responses are generally not sufficient or durable enough to fully eliminate tumors^48^. As such, even though no significant differences in TMB were observed in the two groups in this study, ICI treatment responses may ultimately be shaped by differences in immune cell infiltration within the local tumor microenvironment in the context of different patterns of m^6^A modification. The overall mutation rates in frequently mutated genes were largely similar irrespective of patient m^6^A scores in this study, although mutational frequencies for TP53 and CDKN2A differed significantly between patients with low and high m^6^A scores. TP53 encodes the p53 tumor suppressor protein, and its expression is subject to control mediated by both promoter methylation and post-transcriptional m^6^A mRNA modification such that the silencing of m^6^A methyltransferases can significantly dysregulate p53 signaling activity through changes in gene expression and alternative splicing^5^. The mutation or deletion of TP53 is a major mechanism by which tumors evade immune detection through alterations in the local immune microenvironment via increases in the recruitment of regulatory T cells and the downregulation of MHC-I presentation^51^. How CDKN2A mutations influence ICI responses in different tumors remains somewhat controversial. In renal cell carcinoma patients, for example, mutations in CDKN2A have been linked to reductions in ICI-related clinical benefit^52^, whereas they reportedly had no effect in melanoma^53^. No prior studies have explored the association between CDKN2A mutations and ICI responses in OSCC. As such, these results may suggest that TP53 mutations impact the immune microenvironment through mechanisms related to differences in m^6^A methylation patterns, thereby contributing to differences in how patients respond to ICI treatment. No significant differences in PD-L1 or CTLA-4 expression were observed between patient groups in this study, indicating that m^6^A score may be a more reliable predictor of immunotherapeutic efficacy. As the analyzed datasets did not provide information on microsatellite instability, the relationship between this parameter, m^6^A modification patterns, and OSCC patient outcomes warrants further study.

## Conclusions

In summary, these data highlight the important role that m^6^A modifications play in shaping prognostic outcomes in individuals with OSCC. Differences in these m^6^A modification patterns are important contributors to the observed heterogeneity among patients, and further in-depth analyses of these m^6^A patterns in individual tumors may thus offer new insight into the pathogenesis of OSCC and associated immune cell infiltration within the local tumor microenvironment. These data also offer new insight with the potential to improve clinical responses to immunotherapeutic treatment through the identification of specific tumor immunophenotypes, thereby aiding in the personalized selection of appropriate checkpoint inhibitor regimens with the highest chances of achieving beneficial outcomes in a given patient.

## Methods

### OSCC dataset analyses

Transcriptomic mRNA expression data and corresponding clinical details and somatic mutation data for all OSCC patients included in the TCGA database (https://portal.gdc.cancer.gov/) were downloaded. Diseases included in this dataset included tumors of “other and ill-defined sites in lip, oral cavity and pharynx”, “oropharynx”, “other and unspecified parts of tongue”, “floor of mouth”, “other and unspecified parts of mouth”, “base of tongue”, “gum”, “lip” and “palate”. In total, transcriptomic data in the FPKM (fragments per kilobase per million) format from 340 cancer patients and 32 normal control patients were obtained and transformed into the TPM (transcripts per kilobase million) format using the R “limma” package for targets with known gene symbols. The GSE41613 dataset from the GEO database (https://www.ncbi.nlm.nih.gov/geo/) was also selected for analysis as it contained enough OSCC patients. Clinical information for patients with OSCC in the TCGA and GSE41613 cohorts were combined for univariate Cox analyses and Kaplan–Meier survival analyses, with samples lacking corresponding being excluded from these analyses. Gene-level copy number data for tumor patients were obtained from the following source: https://xena.ucsc.edu/.

### m^6^A regulator-based consensus clustering

After combining data for patients in the selected TCGA and GEO cohorts, the R “ConsensusClusterPlus” package was used to group samples into two m^6^A modification pattern-based clusters according to m^6^A expression levels. In total this clustering was performed based on the expression of 23 m^6^A regulators, including eight writers (METTL3, METTL14, METTL16, RBM15, RBM15B, WTAP, VIRMA, and ZC3H13), two erasers (ALKBH5 and FTO) and 13 readers (YTHDC1, YTHDC2, YTHDF1, YTHDF2, YTHDF3, IGF2BP1, IGF2BP2, IGF2BP3, HNRNPA2B1, HNRNPC, FMR1, LRPPRC, and RBMX).

### Gene set variation analysis (GSVA)

Differences in biological activity in these different m^6^A OSCC patient clusters were explored based on a GSVA enrichment analysis approach performed by the R “GSVA” package and the KEGG pathway set (“c2.cp.kegg.v7.5.1.symbols”) downloaded from http://www.gsea-msigdb.org/. An adjusted p < 0.05 was the threshold for significance.

### Single-sample gene-set enrichment analysis (ssGSEA)

Immune cell infiltration in tumor tissue samples for patients in different m^6^A clusters was explored through a ssGSEA approach. Markers for 23 types of immune cells were analyzed based on prior literature evidence^54^. Enrichment scores were thus generated corresponding to the relative abundance of different populations of infiltrating immune cells.

### Functional enrichment analyses

DEGs were identified based on the intersection of gene expression datasets in the two defined m^6^A clusters using the R “limma” package, with p < 0.001 as the significance threshold. GO and KEGG functional enrichment analyses of these DEGs were then performed, with p < 0.05 as the significance threshold.

### Prognostic m^6^A-related scoring model development

An m^6^A-related scoring model was developed as a tool to predict OSCC patient prognostic outcomes^54^. Initially, samples were stratified into multiple groups based on DEGs expression patterns using an unsupervised clustering approach. Univariate Cox regression analyses were then used to identify genes significantly associated with patient prognosis (p < 0.05). A principal component analysis (PCA) was then used to extract two principal components (PC1 and PC2) as independent variables from these m^6^A clusters. The m^6^A scoring formula was then developed as follows: m6Ascore = ∑(PC1_i_ + PC2_i_), where “i” corresponds to significant prognostic genes.

### Statistical analysis

All statistical analyses were performed using R (V 4.1.2, https://www.r-project.org/) packages downloaded from http://www.bioconductor.org.

### Ethics approval and consent to participate

The patient data in this work were acquired from the publicly available datasets whose informed consent of patients were complete.

## Supplementary Information

Supplementary Information.

## Data Availability

All data used in this work can be acquired from the Gene-Expression Omnibus https://www.ncbi.nlm.nih.gov/geo/, the Cancer Genome Atlas https://portal.gdc.cancer.gov/ and https://xena.ucsc.edu/.

## Abbreviations

CNV: Copy number variation
DEGs: Differentially expressed genes
FPKM: Fragments per kilobase per million
GEO: Gene-Expression Omnibus
GSVA: Gene set variation analysis
ICIs: Immune checkpoint inhibitors
IPS: Immunophenoscore
m^6^A: *N*^6^-Methyladenosine
OS: Overall survival
OSCC: Oral squamous cell carcinoma
PCA: Principal component analysis
PD-L1: Anti-PD-1/programmed death-ligand 1
ssGSEA: Single-sample gene-set enrichment analysis
TCGA: The Cancer Genome Atlas
TMB: Tumor mutation burden
TPM: Transcripts per kilobase million
