# Supplementary material for: Characterization of the m6A regulator-mediated methylation modification patterns in oral squamous cell carcinoma
Source: Sci Rep. 2023 Apr 24;13:6617. doi: 10.1038/s41598-023-33891-9 (PMC10126108; doi:10.1038/s41598-023-33891-9)
Supplement: Supplementary file 1 — Supplementary Information. [file 41598_2023_33891_MOESM1_ESM.pdf]

Additional file Information

Characterization of the m^{6}A regulator-mediated methylation modification patterns in oral squamous cell carcinoma

Lu Pan[1,3,4], He She[2,3,4], Keyi Wang[1,3,4], Wenhui Xia[1,3,4], Haonan Tang[1,3,4], Yuan Fan[1,3,4]* and Jinhai Ye[2,3,4]*

[1]Department of Oral Mucosal Diseases, The Affiliated Stomatological Hospital of Nanjing Medical University, Jiangsu, China

[2]Department of Oral and Maxillofacial Surgery, The Affiliated Stomatological Hospital of Nanjing Medical University, Jiangsu, China

[3]Jiangsu Province Key Laboratory of Oral Diseases, Nanjing Medical University, Jiangsu, China

[4]Jiangsu Province Engineering Research Center of Stomatological Translational Medicine, Jiangsu, China

* Corresponding author

Dr Yuan Fan

Department of Oral Mucosal Diseases, The Affiliated Stomatological Hospital of Nanjing Medical University. 136# Hanzhong Road, Nanjing, Jiangsu 210000, China

E-mail: fanyuan@njmu.edu.cn

Dr Jinhai Ye

Department of Oral and Maxillofacial Surgery, The Affiliated Stomatological Hospital of Nanjing Medical University. 136# Hanzhong Road, Nanjing, Jiangsu 210000, China

E-mail: yejinhai@njmu.edu.cn

Additional file Figures

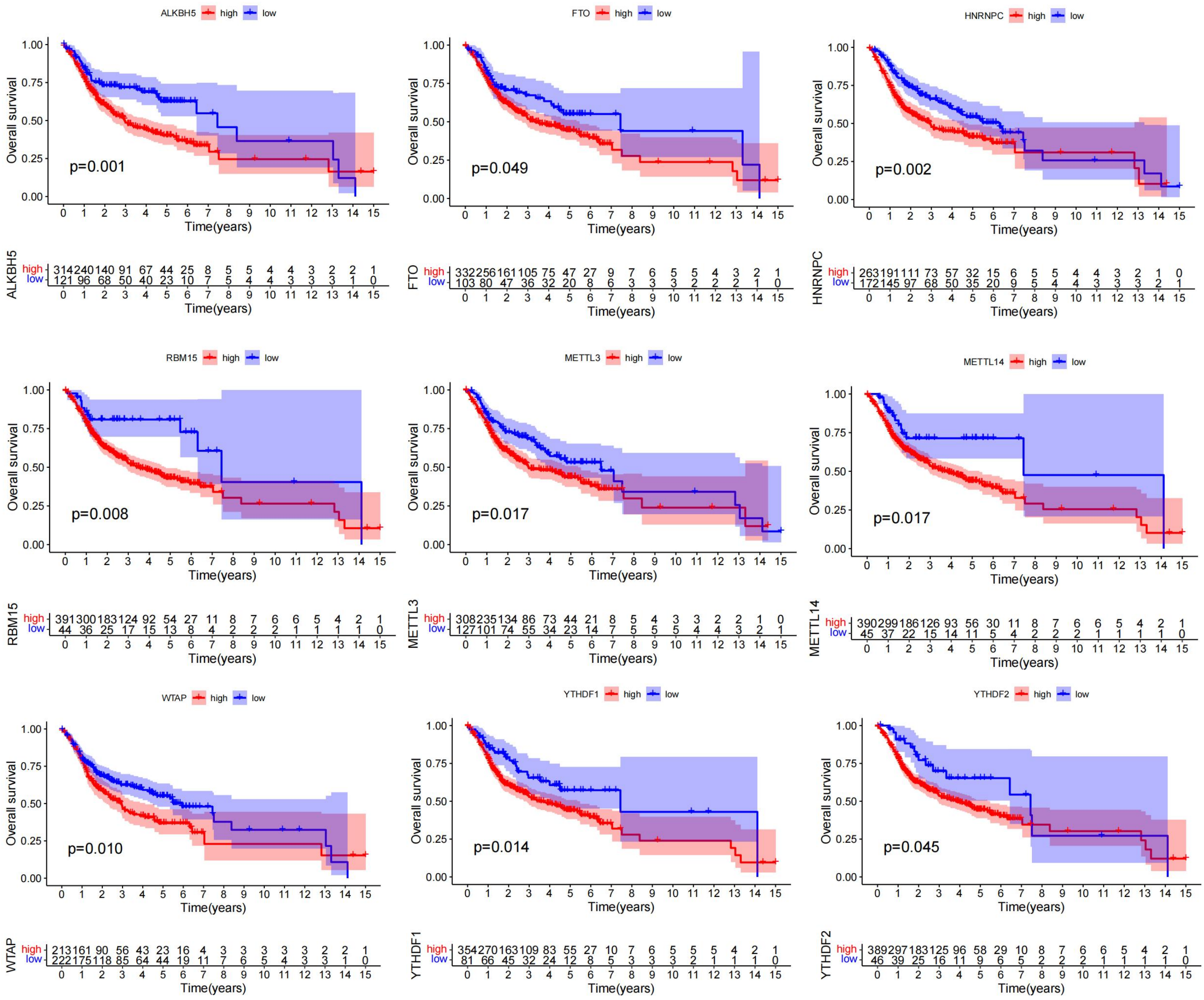

Figure S1. Kaplan-Meier survival analysis of m^{6}A regulators between the high-expression group and the low-expression group using clinical information of OSCC patients in TCGA and GSE41613 cohort. *P* value less than 0.05 was statistically significant.

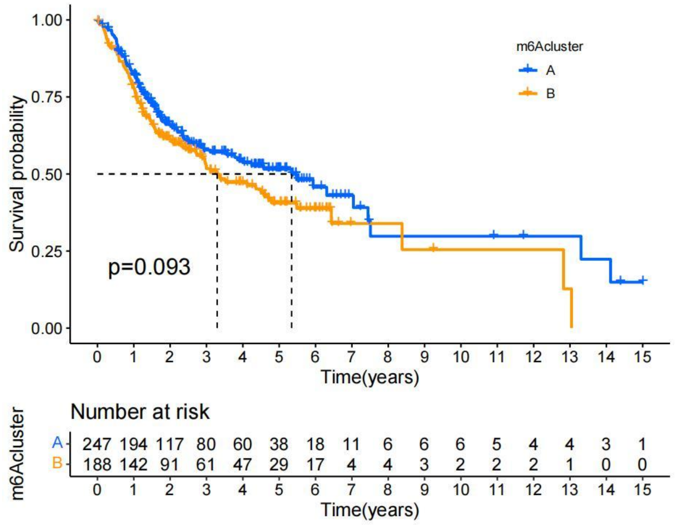

Figure S2. Kaplan-Meier survival analysis of OSCC patients in different m^6A clusters. Cluster A, blue curve; Cluster B, yellow curve. Log-rank $p = 0.093$ showed no significant survival difference between two m^6A clusters.

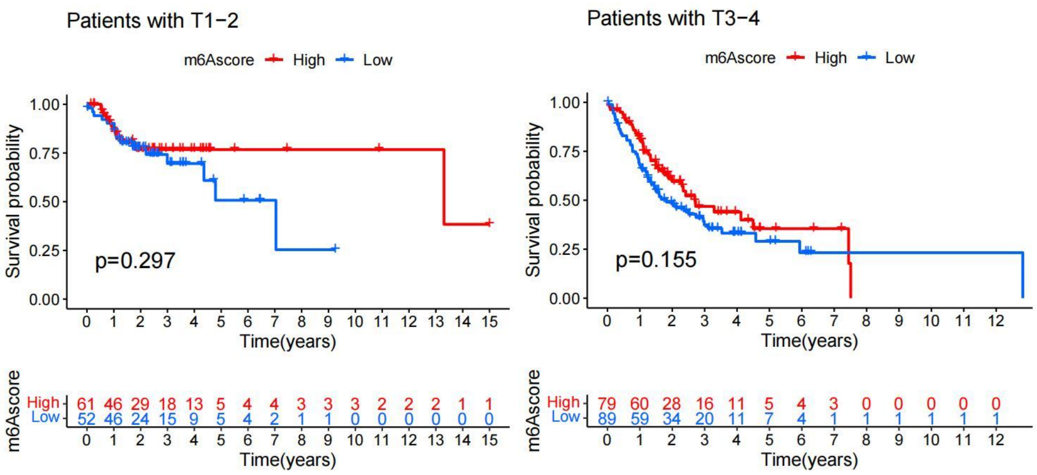

Figure S3. Kaplan- Meier survival analysis of OSCC patients with specific clinical stages (T1-2 and T3-4) in different m6Ascore groups. Low-m6Ascore group, blue; High- m6Ascore group, red. There was no statistical significance between the low- m6Ascore group and the high- m6Ascore group whether the patients were in the T1-2 group or the T3-4 group.

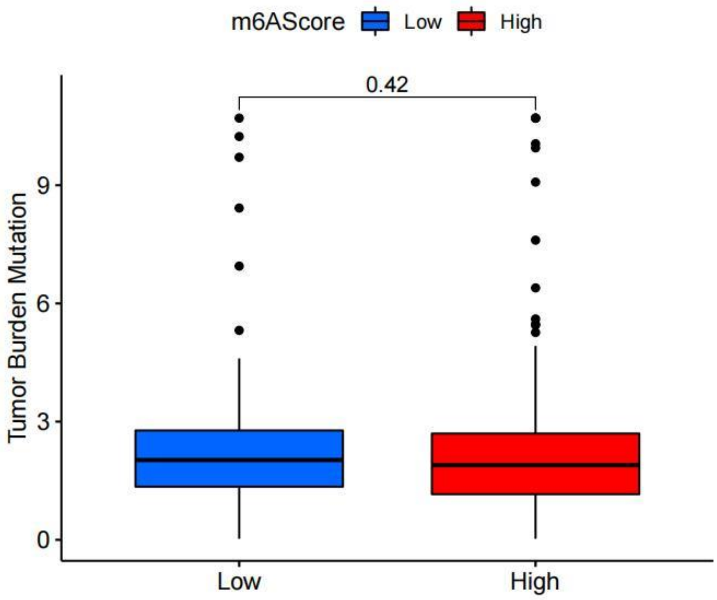

Figure S4. Differences in TMB between the low-m6Ascore group and the high-m6Ascore group. There were no significant differences between the two groups with $p = 0.42$.

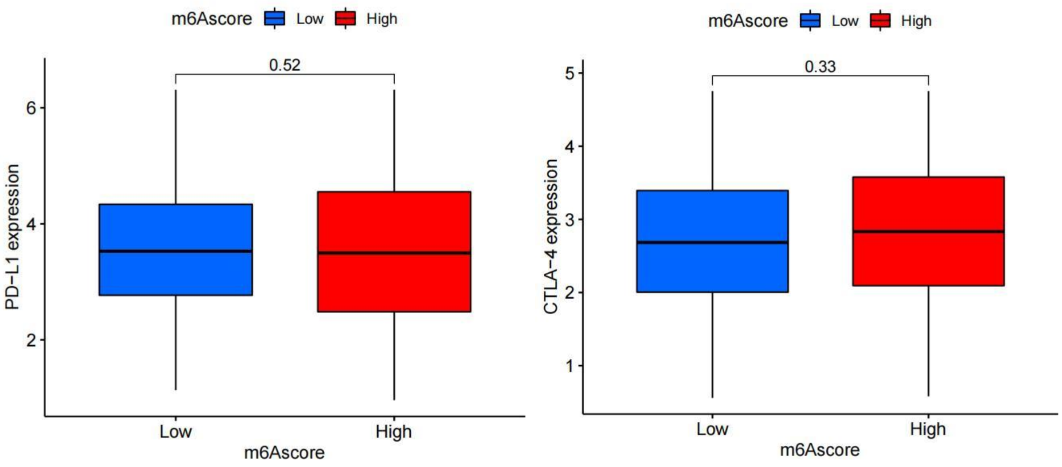

Figure S5. Differential expression of *PD-L1* and *CTLA-4* between the low-m6Ascore group and the high-m6Ascore group. No significant differences were showed in the expression of either *PD-L1* (p = 0.52) or *CTLA-4* (p = 0.33).

Additional file Tables

Table S1. Univariate Cox regression analysis of DEGs between two distinct m^6A methylation modification patterns.

id	HR	HR.95L	HR.95H	*p* value
EFCAB11	1.946254667	1.433090713	2.643173384	2.01E-05
PCMT1	1.961544036	1.434074461	2.68302317	2.49E-05
TEX19	1.978735201	1. 197117387	3.270684261	0.007775703
GRPR	1.979721757	1. 110542129	3.529175647	0.020589303
WDFY3-AS2	2.095564408	1.339210045	3.279089939	0.001201501
CCDC43	2. 122907287	1.50470711	2.995091417	1.81E-05
HOTTIP	2.673356944	1.068659164	6.687667678	0.035561512
OTOGL	3.235670894	1.403334002	7.460494883	0.005869774
EYS	4. 11579832	1.372695489	12.34053433	0.011556982
PRKG1-AS1	7.65816451	3.234342661	18. 13273664	3.67E-06
PSMD6-AS2	0.542486551	0.33069847	0.889909344	0.015442724
SH3BP5L	0.653947947	0.485001223	0.881746059	0.005348353
SCN8A	0.673815166	0.488854684	0.928756322	0.015891792
CEACAM21	0.697879525	0.549521199	0.886291253	0.003179081
ZNF823	0.707407898	0.558850826	0.895455301	0.004000914
SZT2	0.709656947	0.544908043	0.92421646	0.01093762
NAGK	0.71119248	0.549345753	0.920722042	0.009684045
EML6	0.72346702	0.546623225	0.9575234	0.02360647
ZNF266	0.73609384	0.591803509	0.915564259	0.005915883
PCF11	0.74332574	0.598586691	0.923062881	0.007263542

HR value less than 1 is low risk, greater than 1 is high risk; $p < 0.001$ was statistically significant. The table shows the top 10 low risk and top 10 high risk genes.

Table S2. Top ten high-frequency mutated genes based on masked somatic mutation database of OSCC patients from TCGA.

Gene	Sample Number
TP53	313
TTN	175
FAT1	104
CDKN2A	89
MUC16	85
PIK3CA	81
CSMD3	81
NOTCH1	78
SYNE1	75
LRP1B	69
